# Alternative lengthening of telomeres is not synonymous with mutations in ATRX/DAXX

**DOI:** 10.1038/s41467-021-21794-0

**Published:** 2021-03-10

**Authors:** Alexandre de Nonneville, Roger R. Reddel

**Affiliations:** 1 Cancer Research Unit, Children’s Medical Research Institute, Faculty of Medicine and Health, University of Sydney, Westmead, NSW, Australia; 2 Aix-Marseille Univ, CNRS, INSERM, Institut Paoli-Calmettes, CRCM, Marseille, France

**Keywords:** Cancer genetics, Cancer genomics

**Arising from** Lina Sieverling et al. *Nature Communications* 10.1038/s41467-019-13824-9 (2020)

The PCAWG Consortium has recently released an integrative re-analysis of a large set of tumor whole genome sequence (WGS) data from 2658 cancer patients across 38 different primary tumor sites^1^. In a companion paper, Sieverling et al. built a random forest classifier for the telomere maintenance mechanism (TMM) by regarding truncating ATRX or DAXX alterations, referred to as ATRX/DAXX^trunc^, vs. TERT modifications (TERT^mod^; i.e., promoter mutations ±  amplifications ±  structural variations), as indicators of alternative lengthening of telomeres (ALT) vs. telomerase^2^. We show here that equating ATRX/DAXX^trunc^ and TERT^mod^ with ALT and telomerase, respectively, results in TMM predictions which do not correlate well with TMM assay data. Although ATRX/DAXX^trunc^ mutations are associated with TMM, most tumors do not harbor them and they are heterogeneously distributed in ALT-positive (ALT + ) tumors of different types, as are TERT^mod^ in telomerase-positive tumors^3–6^, making these mutations an insufficient basis for building a classifier in a large-scale pan-cancer study.

Here, we provide a new analysis of the PCAWG data, based on C-circle assay (CCA)^7^ data that are available for a subset of these tumors^3^. We show that the Sieverling et al. score overestimates the proportion of ALT associated with ATRX/DAXX^trunc^ and misclassifies ALT tumors when these mutations are absent. We also show some telomere variant repeats (TVR) correlate with ATRX/DAXX^trunc^ mutations, regardless of TMM. Finally, we propose a new classifier to identify ALT tumors in the PCAWG cohort.

WGS data have been used as a means to assess telomere content and analyze TMM^3^. To our knowledge, only one previous genomic study generated an ALT-probability score based on ALT assay data. The CCA appears to be a reliable marker of the presence of ALT activity in cancers^7^, notwithstanding cell line studies showing that quantitative CCA data do not always correlate with the amount of ALT activity (e.g., ref. ^8^), highlighting the need for further biological studies to decipher the origin of C-circles and their functional relationship with the ALT mechanism. Lee et al.^3^ used the CCA to determine ALT status of 167 pancreatic neuroendocrine tumors (PaNET) and melanomas, and then applied machine learning to features including total telomeric and TVR content to develop an ALT classifier with an accuracy of 91.6%. The classifier was then applied to WGS data from 908 additional tumors, mostly from The Cancer Genome Atlas (TCGA) dataset. Of the total of 1075 tumors studied by Lee et al.^3^, 703 were included in the PCAWG study, and CCA data are available for 114 of these (melanoma, *n* = 46 and PanNET, *n* = 68). A comparison of the Sieverling and Lee scores revealed a poor correlation (*r* = 0.101 [95% CI 0.025–0.17], Spearman correlation; Supplementary Fig. 1). We then compared the CCA data with Sieverling’s score. The latter identified only 64.5% of CCA-positive tumors as ALT-high probability and misidentified CCA-negative ATRX/DAXX^trunc^ tumors (Fig. 1). Also, the only CCA-positive TERT^mod^ tumor was identified as ALT-low probability by the Sieverling score.

**Fig. 1:**
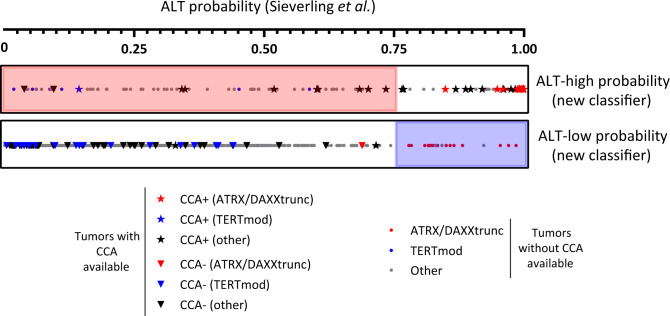
Comparison of the ALT-probability score of Sieverling et al.^2^, with the new classifier proposed in this article. The 2497 patients described in Sieverling et al. are divided here into two groups: ALT-high probability (upper bar) and ALT-low probability (lower bar) according to the new classifier. Within the two bars, each patient is represented by a symbol placed on a scale from 0 to 1 according to its Sieverling ALT-probability score. Star and inverted triangle symbols represent patients whose tumors were C-circle assay (CCA) positive or negative, respectively^3^, and the dots represent patients for whom CCA data are unavailable. Red and blue symbols correspond to patients with ATRX/DAXXtrunc and TERTmod alterations, respectively^2^. The pink and blue shading highlights the tumors for which the two scores were discordant: the pink shading in the upper box indicates tumors with ALT-high probability (new classifier), that were ALT-probability <0.75 (ALT-low) by Sieverling’s score; the blue shading in the lower box indicates tumors with ALT-low probability (new classifier), that were ALT-probability >0.75 (ALT-high) by Sieverling’s score.

Sieverling et al.’s analysis of the PCAWG data identified an association between prevalence of TTCGGG singletons and ALT, and a more pronounced enrichment of the TGAGGG TVR than previously noted. Our re-analysis showed that TTCGGG, TGAGGG, TTTGGG, and TTGGGG singleton distributions are similar among ATRX/DAXX^trunc^ tumors, regardless of CCA result (Fig. 2). In contrast, in CCA-positive tumors, there were statistically significant differences in TTCGGG and TGAGGG distributions between tumors with and without ATRX/DAXX^trunc^.

**Fig. 2:**
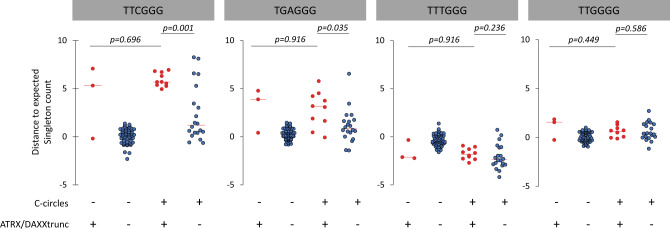
Distance to the expected singleton repeat count in tumors with negative C-circle assay, with (*n* = 3), or without (*n* = 58) ATRX/DAXXtrunc, and in tumors with positive C-circle assay with (*n* = 10), or without (*n* = 20) ATRX/DAXXtrunc. The center (horizontal) red line of the scattergrams is the median. *p* < 0.05 was considered as significant; Kolmogorov–Smirnov tests, two-tailed.

ATRX is a SWI/SNF-like chromatin remodeling protein that binds to G-rich tandem repeats^9^. Once recruited, ATRX cooperates with histone chaperone DAXX in replication-independent deposition of the histone variant H3.3. Loss of ATRX/DAXX function causes defects in multiple cellular processes, including defective sister chromatid cohesion and telomere dysfunction but in some cellular contexts is not sufficient by itself to induce ALT^10^. Our data suggest that ATRX/DAXX^trunc^ (rather than the ALT mechanism per se) could also play a role in TVR distribution. This observation could provide additional insights regarding the mechanisms involved in ALT promotion in the context of ATRX/DAXX^trunc^ in specific tumor types. TVR are associated with genomic instability, and could facilitate telomere spatial reconfiguration and affect telomere binding affinity^11,12^. Although histones are non-specific DNA binding proteins, nucleosome formation could potentially be influenced by telomeric DNA sequences and their structural properties^13^.

The international effort that enabled release of the PCAWG data set has provided a major new resource for seeking new insights into cancer, including the ALT pathway, which represents a potentially valuable, currently unexploited, target for anti-cancer therapies. Understanding more precisely how telomeres are maintained in cancer will shed light on replicative immortality and telomeric DNA damage response mechanisms. To develop a classifier, based on CCA data as an indicator of ALT, we searched for a signature associated with CCA status within the genomic features provided by the Telomere Hunter WGS tool^2^. Of the eight features used by Sieverling et al. in their random forest classifier, five were retained after Akaike information criterion stepwise regression analysis: telomere content (tumor/control log2 ratio) and the distance of TTTGGG, TTCGGG, TTGGGG, and GTAGGG singletons from their expected occurrence. A classifier was then built from these five features, which permitted definition of two groups as “high-probability” and “low probability”, using different combinations of the PanNET and melanoma datasets for training and testing. A 100% success rate was achievable when considering only one tumor type (i.e., using PanNET or melanoma for both the learning and the validation sets). Our final classifier, built using the whole cohort of patients with CCA data, has an accuracy of 93.86% (93.55% specificity and 93.98% sensitivity). This underlines the need for caution when extrapolating a classifier built on a specific tumor type to others. When applied to the 2497 PCAWG patients, our classifier identified 200 tumors with high probability of ALT (Supplementary data 1), and their reported distribution across different histological types (leiomyosarcomas 73%, osteosarcomas 63%, liposarcomas 53%, and low grade gliomas 29%) is consistent with previous reports^5^. In contrast, 27% of ATRX/DAXX^trunc^ tumors (*n* = 17/64) were classified as ALT-low probability, and 6% of TERT^mod^ tumors (*n* = 15/269) as ALT-high probability (Fig. 1).

In conclusion, the Sieverling et al. score is an ATRX/DAXX^trunc^ vs. TERT^mod^ classifier rather than a predictor of ALT. The view that ATRX/DAXX loss is essentially equivalent to the presence of ALT activity may apply only to specific types of tumors, in specific genomic or epigenetic contexts, as suggested by recent cell line based studies^14,15^. Adoption of this view as a generalization across cancer types may divert attention from the need to identify alternative molecular actors involved in TMM. The classifier based on telomeric content and TVR distributions we provide here is more accurate as judged by C-circles, a hallmark of ALT, for PanNETs and melanomas, the tumor types on which it was trained. Although its predictions for other tumor types are consistent with the reported prevalence of ALT, this classifier should be applied with caution, given the apparent tumor type specificity of TVR distribution.

## Methods

Sieverling et al.^2^ and Lee et al.^3^ ALT-probability scores were compared using Spearman’s correlation. Distances to the expected singleton repeat count distributions^2^ in tumors with negative CCA^3^, with or without ATRX/DAXXtrunc^2^, and in tumors with positive CCA^3^ with, or without ATRX/DAXXtrunc^2^ were compared using two-tailed Kolmogorov–Smirnov tests at the 5% level of significance. To develop a classifier, based on CCA data^3^ as an indicator of ALT, we searched for the best combination signature associated with CCA status^3^ within the genomic features and telomere content determined by Sieverling et al. with the Telomere Hunter WGS tool^2^ (telomere content tumor/control log2 ratio, telomere insertion count, breakpoint count, and singleton distributions), using Akaike information criterion stepwise regression analysis. Statistical analyses were carried out using R version 3.6.2 (R Foundation for Statistical Computing).

### Reporting summary

Further information on research design is available in the Nature Research Reporting Summary linked to this article.

## Supplementary information

Supplementary Information

Supplementary Data 1

Reporting Summary

## Data Availability

This Matters Arising is a non-interventional, retrospective analysis and commentary of published data, publicly available within the Nature Communications paper “Sieverling et al. Genomic footprints of activated telomere maintenance mechanisms in cancer. *Nat. Commun*. **11**, 1–13 (2020).”^2^, and the Nucleic Acids Research paper “Lee et al. Telomere sequence content can be used to determine ALT activity in tumors. *Nucleic Acids Res*. 46, 4903–4918 (2018).”^3^ Genomic features and telomere content determined by Sieverling et al., and the Sieverling et al. score, are available in the Sieverling et al.^2^ supplementary data 1 file. C-circle data, referenced by Lee et al.^3^, and the Lee et al. score, are available in the Lee et al.^3^ supplementary data file. Aligned PCAWG read data in BAM format are also available at the European Genome Phenome Archive (EGA; https://www.ebi.ac.uk/ega/search/site/pcawg under accession number EGAS00001001692). In addition, all open-tier PCAWG genomics data, as well as datasets used for PCAWG analysis, can be downloaded from the ICGC Data Portal at http://docs.icgc.org/pcawg/data/. The software TelomereHunter used by Sieverling et al.^2^ for telomeric in silico analysis is available from https://www.dkfz.de/en/applied-bioinformatics/telomerehunter/telomerehunter.html. The core computational pipelines used by the PCAWG Consortium for alignment, quality control, and variant calling are available to the public at https://dockstore.org/search?search=pcawg under the GNU General Public License v3.0, which allows for reuse and distribution.
