# Supplementary material for: Alternative lengthening of telomeres is not synonymous with mutations in ATRX/DAXX
Source: Nat Commun. 2021 Mar 10;12:1552. doi: 10.1038/s41467-021-21794-0 (PMC7946928; doi:10.1038/s41467-021-21794-0)
Supplement: Supplementary file 1 — Supplementary Information [file 41467_2021_21794_MOESM1_ESM.pdf]

Matters Arising

Alternative Lengthening of Telomeres is not synonymous with mutations in ATRX/DAXX

de Nonneville *et al.*

Supplementary Information

Supplementary Figure 1: Correlation between Sieverling *et al.* and Lee *et al.* ALT-probability scores.

Supplementary Figure 1

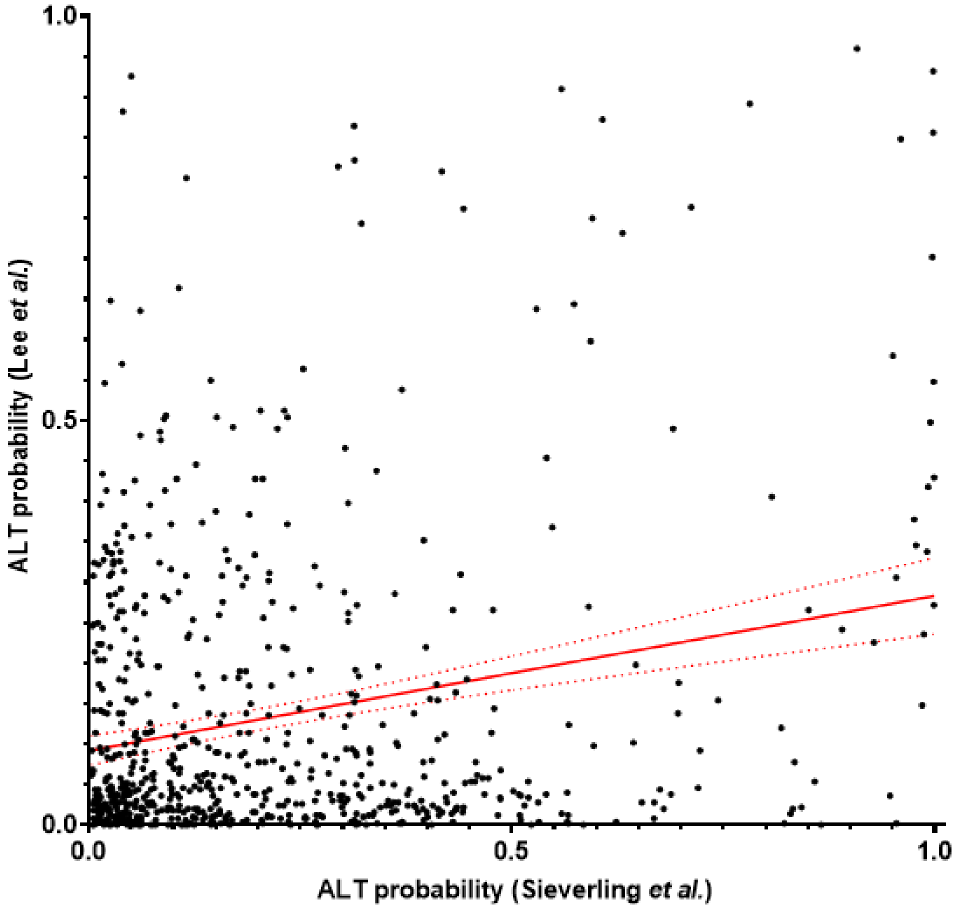

Supplementary Figure 1. Correlation between Sieverling *et al.*[2] and Lee et *al.*[3] ALT-probability scores. Spearman r=0.101 [95%CI 0.025-0.17].
